# Electromagnetic Nanocoils Based on InGaN Nanorings

**DOI:** 10.3390/nano15030245

**Published:** 2025-02-05

**Authors:** Ziwen Yan, Peng Chen, Xianfei Zhang, Zili Xie, Xiangqian Xiu, Dunjun Chen, Hong Zhao, Yi Shi, Rong Zhang, Youdou Zheng

**Affiliations:** 1 Key Laboratory of Advanced Photonic and Electronic Materials, School of Electronic Science and Engineering, Nanjing University, Nanjing 210093, China; dg21230063@smail.nju.edu.cn (Z.Y.); 602022230055@smail.nju.edu.cn (X.Z.); xzl@nju.edu.cn (Z.X.); xqxiu@nju.edu.cn (X.X.); djchen@nju.edu.cn (D.C.); zhaohong@nju.edu.cn (H.Z.); yshi@nju.edu.cn (Y.S.); ydzheng@nju.edu.cn (Y.Z.); 2 School of Electronic Science and Technology, Xiamen University, Xiamen 361005, China; rzhang@nju.edu.cn

**Keywords:** InGaN, NSAE, nanoring, electromagnetic nanocoils

## Abstract

Energy issues, including energy generation, conversion, transmission and detection, are fundamental factors in all systems. In micro- and nanosystems, dealing with these energy issues requires novel nanostructures and precise technology. However, both concept and setup are not well established yet in the microsystems, especially for those at the nanometer scale. Here, we demonstrate electromagnetic nanocoils with 100 nm diameters based on uniform and periodic InGaN nanoring arrays grown on patterned GaN surfaces using nanoscale selective area epitaxy (NSAE). We observed stronger photoluminescence from the periodic InGaN nanoring arrays compared to the non-uniform InGaN nanorings, which indicates good crystal quality of the InGaN nanostructure with the NSAE. Based on this kind of nanostructure, electromagnetic induction from the nanorings is detected through the rebound movement of high-energy electron diffraction patterns that are influenced by a modulated external magnetic field. Our results clearly show the generation of an inductive current and internal magnetic field in the nanorings. We anticipate this kind of nanostructure to be a potential key element for energy conversion, transfer and detection in nanosystems. For example, it could be used to fabricate microtransformers and micro- and nanosensors for electromagnetic signals.

## 1. Introduction

Low-dimensional semiconductor structures such as quantum dots, nanorings and nanowires give rise to new physical phenomena that have been applied in optoelectronic and electronic devices with novel functionalities, such as quantum dot (QD) laser diodes (LDs) [1] and single-electron transistors [2]. In particular, semiconductor nanorings are more complex quantum systems. On the one hand, nanoring structures provide us with a unique opportunity to study rings at true quantum limits, and this unique nanostructure has attracted intense interest in the quantum interference phenomenon and excitonic Aharonov–Bohm (AB) effect [3,4] in transport and optical properties recently [5,6,7,8,9,10,11,12,13,14,15]. On the other hand, this structure also provides a current path in nanoscale systems, in which a current can be induced, and furthermore, a magnetic field can be generated only in the inner hole. This kind of semiconductor nanocoil is significant for electromagnetic signal generation, detection and energy conversion in integrated nanosystems for optoelectronic and biological applications.

To date, the formation of semiconductor nanorings is often through self-organized processes, mostly controlled by the Stranski–Krastanow (S–K) growth mode [16]. Using this method, semiconductor nanorings were fabricated [5,16,17,18,19,20,21,22]. Some researchers control structure morphology to form nanorings by modulating adatom migration rate, controlling surface free energy balance [23] or using lithographic techniques [7]. However, the random distribution of ring sizes and locations usually occurs. Besides the S–K mode, some researchers developed a few unique methods to create nanorings. By using polar surface-induced spontaneous self-coiling processes, freestanding single-crystal complete nanorings of zinc oxide were formed [24]. Hollow nanocrystals can be synthesized through a mechanism analogous to the Kirkendall Effect, in which pores form because of the difference in diffusion rates between two components in a diffusion couple [25].

In order to achieve uniform and periodic structure arrays, many different surface processing methods were carried out to promote nucleation at expected sites, including surface state control [26,27,28], shallow modulated substrate surfaces [29,30] and nanoscale selective area epitaxy (NSAE) on patterned surfaces [31,32,33,34]. By using the NSAE, we have produced uniform and periodic nanodot/nanoring/nanowire arrays, which have mean lateral sizes of less than 100 nm with standard deviations of only 2.94% for the nanodots/nanorings [35,36].

Here, we present the work on electromagnetic induction from the long-range ordered InGaN nanoring arrays on the patterned GaN surface. The nanopatterns were defined by electron-beam lithography (EBL). The InGaN nanoring arrays were grown using NSAE in metalorganic chemical vapor deposition (MOCVD). The electromagnetic induction from the nanorings was detected through the rebound movement of reflective high-energy electron diffraction (RHEED) patterns in a modulated external magnetic field, based on an analysis of the nature of the beam shift.

## 2. Materials and Methods

To fabricate the nanostructures, first, a semi-insulating GaN film was grown on a c-plane sapphire, up to a thickness of 2 μm. Next, 80 nm SiO_2_ was deposited onto the GaN film and then processed with EBL (JBX-9300, JEOL Ltd., Tokyo, Japan) to create nanopatterns in the SiO_2_. The fabrication details have been described elsewhere [34,35,36] (Supplementary Materials: Figure S1). InGaN was then grown on the patterned SiO_2_/GaN in the Emcore D-125 MOCVD reactor (Veeco, Plainview, NY, USA) at 750 °C, resulting in the formation of nanodots or nanorings, depending on the pattern sizes and growth durations. The nominal thickness of the InGaN was about 10 nm. On top of the InGaN, another 10 nm GaN cap layer was grown. Finally, the samples were dipped in diluted hydrofluoric acid to remove all SiO_2_ to assess the sample morphology. The samples were investigated by means of scanning electron microscopy (SEM, JSM-7401F, JEOL Ltd., Tokyo, Japan), continuous wave (CW) photoluminescence (PL, KIMMON 325 nm He-Cd laser, Kimmon Koha Co., Ltd., Tokyo, Japan and inVia microscope, Renishaw plc, Gloucestershire, UK) at low temperatures and RHEED (RHEED-30, STAIB INSTRUMENTS, Inc., Williamsburg, VA, USA) at 200 °C.

**Figure 1:**
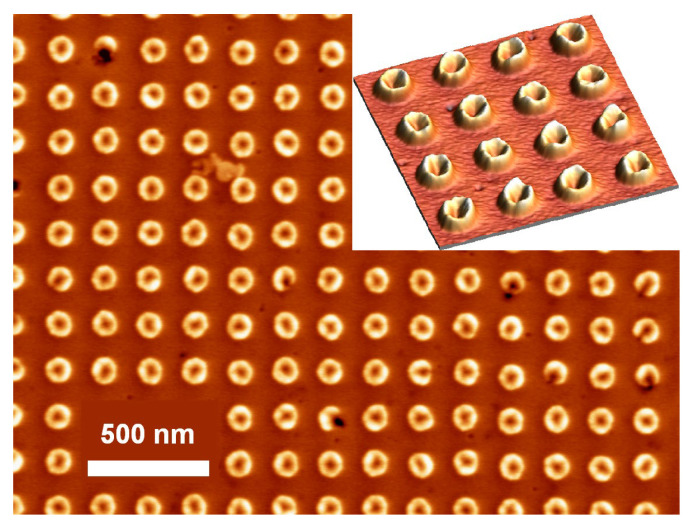
SEM image of the InGaN nanoring arrays grown on the patterned GaN surface. The nanorings are around 100 nm in diameter and are spaced by 200 nm. The inset is a three-dimensional view of a few nanorings.

A typical SEM image of the InGaN nanoring arrays grown on the patterned GaN surface is shown (Figure 1). The nanorings are about 100 nm in diameter and are spaced by 200 nm. The inset is a three-dimensional view of a few nanorings. In this sample, the InGaN nanorings are well developed. The InGaN nanorings have very uniform sizes and regular symmetry. The standard deviation of the nanorings in diameter is around 2.94%.

## 3. Results

**Figure 2:**
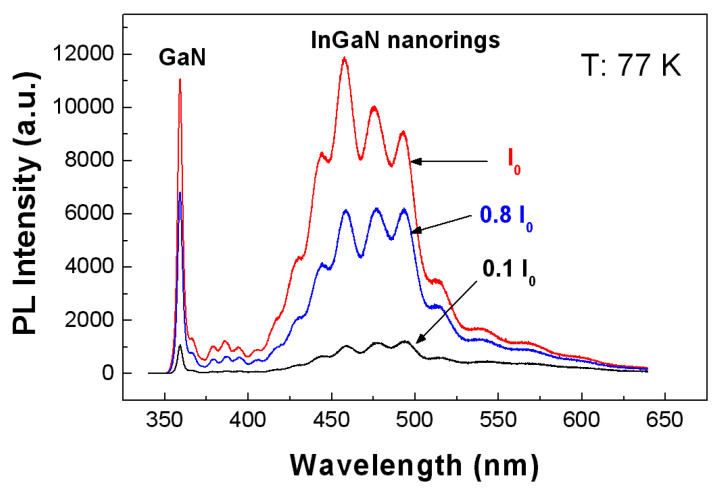
Low-temperature (77 K) PL spectra from the InGaN nanoring arrays with 100 nm diameters under different excitation levels of 0.1 I_0_, 0.8 I_0_ and I_0_, where I_0_ is the highest excitation power.

PL spectra from the 100 nm InGaN nanoring arrays were obtained (Figure 2) under different excitation levels of 0.1 I_0_, 0.8 I_0_ and I_0_, where I_0_ is the highest excitation power of around 1.2 × 10^3^ W/cm^2^. The excitation source is a 325 nm He-Cd CW laser. The emission peak around 355 nm is the band-edge exciton luminescence of GaN, while the emission near 380 nm originates from donor–acceptor pair (DAP) luminescence. The broad emission spectrum from 460 to 500 nm comes from the nanorings. It is seen that the emission from the nanorings is broad. The fringes in the spectra are caused by light interference in the epilayer. Clearly, the broadening is not due to the size non-uniformity, but due to the non-uniform indium composition across the ring. The ring-shaped profile indicates the different growth rates between the edge area and center area in those patterned holes. Furthermore, the indium composition is influenced by the growth rate. A higher growth rate induced higher indium incorporation efficiency in this experiment. As shown in Figure 2, by increasing excitation power, the emission at the shorter wavelength part (around 460 nm) was enhanced more than the part in the longer wavelength range (around 500 nm), which indicates more excitons recombining at higher energy levels under higher excitation power. Due to the large photo-energy difference between 460 nm (2.70 eV) and 500 nm (2.48 eV), this photo-energy increment cannot be fully explained by the band-filling effect. So, it means that the indium composition is not uniform in nanorings. According to the growth rate difference in a nanohole, it is deemed that a higher growth rate at the edge area results in a higher indium composition. The indium accumulation effect at the edge area can be directly observed in the growth with a larger selective area (Supplementary Materials: Figure S2). Thus, these InGaN structures not only have ring-shaped profiles in their geometric image but also a ring-shaped potential in their energetic picture.

It is well known that normal InGaN grown with epitaxy is unintentionally an n-type semiconductor. The InGaN nanorings are also not exceptional and have free electrons with a concentration of 10^18^~10^19^/cm^3^, estimated from InGaN epilayers at room temperature [37,38]. Namely, there should be a few hundred free electrons in a single InGaN nanoring. Once these electrons flow along the ring, there will be an additional magnetic field generated by the ring current. To observe electromagnetic induction from the nanorings, we plan to create the ring current by modulating the external magnetic field.

The electromagnetic induction from the InGaN nanorings was measured by RHEED in a high vacuum chamber (~10^−9^ Torr). The movement of the RHEED patterns was recorded by RHEED monitoring software. The sample was mounted on a Molybdenum holder equipped with a heater at the backside. Because the heater was a wreathed filament, it could provide a modulated external magnetic field by flowing a DC current. To confirm the environment was clean for the RHEED measurement, i.e., without any other source to generate a magnetic field, a bare GaN/sapphire sample was used for reference.

**Figure 3:**
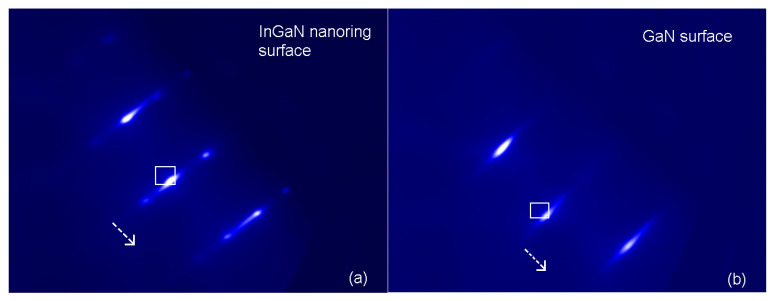
RHEED patterns from (**a**) InGaN nanoring surface and (**b**) bare GaN surface without external magnetic field. Spotty patterns are observed on the InGaN nanoring surface and streaky patterns are observed on the bare GaN surface. The white arrows indicate the movement of these patterns under a magnetic field. The white boxes indicate the areas in which the time-dependent intensity is recorded when the magnetic field is applied.

After desorption of all impurities by keeping the sample in the high vacuum chamber for 24 h, the samples were heated to 200 °C for RHEED. The RHEED patterns from the InGaN nanoring surface and the bare GaN surface were obtained, respectively (Figure 3a,b) without an external magnetic field. As expected, spotty patterns were observed on the InGaN nanoring surface (Figure 3a), while streaky patterns were observed on the bare GaN surface (Figure 3b). When an external magnetic field was exerted, these patterns shifted in the direction indicated by the white arrows in Figure 3. Obviously, the shifting of the patterns was decided by how the external magnetic field was altered. In order to record how the patterns shifted, we chose to record the time-dependent light intensity of a pattern in the selected area, as indicated by the white box in Figure 3. When the pattern moved away, the light intensity dropped. However, if there was any undulation during the movement of the pattern, the intensity was recorded. In this experiment, the external magnetic field was modulated with the heater current, which switched between 0 and 17 A.

**Figure 4:**
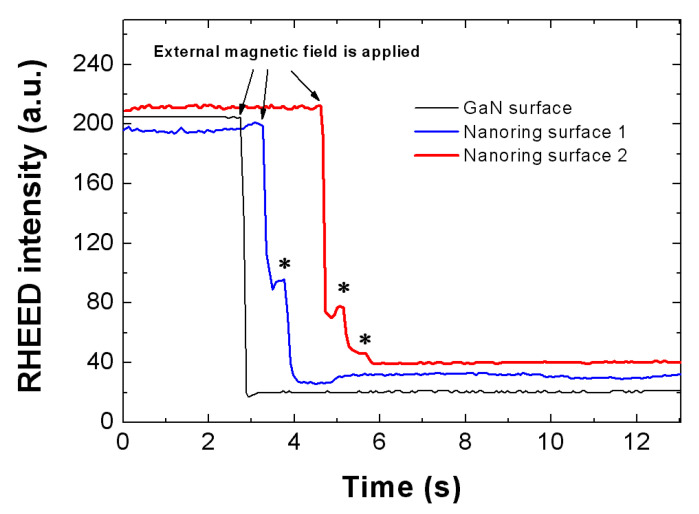
Intensity change in the monitored areas shown in Figure 3 when the external magnetic field is applied. On the bare GaN surface, the intensity drops monotonously to the background value, which indicates a straightforward shift in the pattern. On the nanoring surface, the intensity shows small rebounds during its decrease process, which indicates the return movement of the pattern.

The intensity change in the presence of the external magnetic field was recorded (Figure 4). For the bare GaN reference sample, the intensity dropped monotonously to the background value, which indicates that the bare GaN did not generate any induced magnetic field. This can be easily understood since the GaN is semi-insulating and does not have any ring-shaped potential to confine the movement of electrons. However, the intensity evolvement is different for the InGaN nanoring surface—there were several rebounds during the intensity drop, as marked by the stars in Figure 4. These rebounds indicate that the patterns were not monotonously shifting in one direction; instead, the patterns were pulled back a little during the shift. This indicates the existence of an additional magnetic field generated during the process. The only source for the new magnetic field was the InGaN nanoring arrays. A ring current was first induced in the InGaN nanorings during the increase/decrease in the external magnetic field. In turn, the ring current produced the new magnetic field. Because the increase/decrease in the external magnetic field was not linear, the ring current density and the induced magnetic field intensity varied during the process. The direction of the induced magnetic field could be the same or opposite to the external magnetic field. Indeed, we observed several rebounds during one shift, which indicates the variation in the induced magnetic field. For the whole process of the shift, please refer to the video clips attached in the Supplementary Materials. This phenomenon proves the electromagnetic induction from the InGaN nanorings, acting as electromagnetic nanocoils. As is well known, the electron concentration in semiconductors is more than three orders of magnitude lower than that in conventional metals, resulting in a lower induced magnetic field in this experiment.

**Figure 5:**
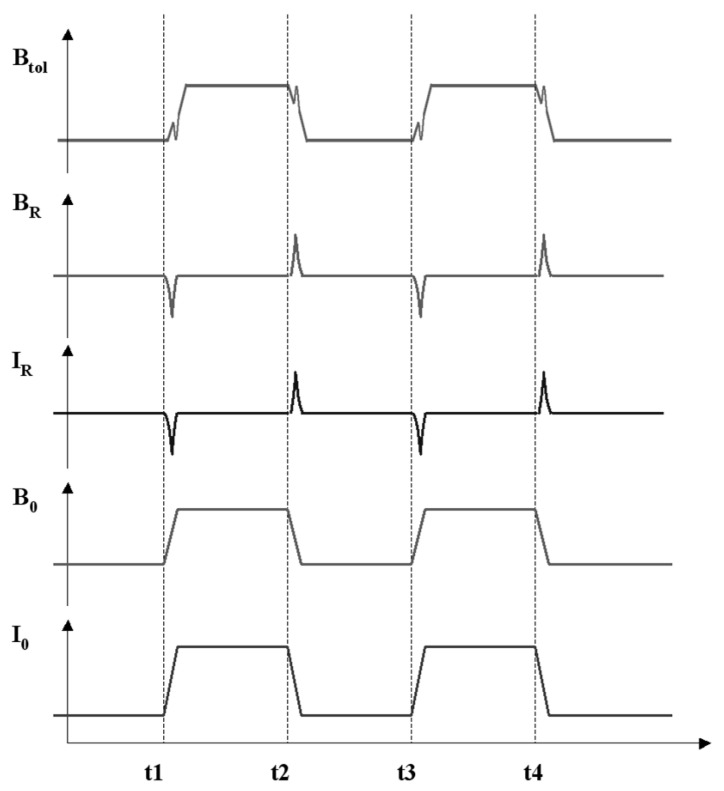
Schematic description of the electromagnetic induction process. I_0_ is the DC current through the heater. B_0_ is the external magnetic field, which is modulated by turning I_0_ “on” and “off”. I_R_ is the inductive ring current in the InGaN nanorings during the changing of B_0_. B_R_ is the inductive magnetic field generated by the I_R_. B_tol_ is the total magnetic field, which is the vector sum of B_0_ and B_R_.

The detailed process is schematically described (Figure 5). The external magnetic field (B_0_) is modulated by turning the DC current (I_0_) of 17 A on and off through the heater. A linear variation of B_0_ is assumed. Due to the ring-shaped InGaN nanostructure existing in the magnetic field, an inductive ring current (I_R_) will be induced in the nanorings during the appearance and disappearance of B_0_. Furthermore, the inductive ring current, I_R_, generates an additional magnetic field (B_R_) that can be the same or opposite to B_0_. Thus, in this system, the total magnetic field (B_tol_) is the vector sum of B_0_ and B_R_ (Figure 5). The movement of the RHEED patterns embodies the behavior of the total magnetic field in this system. Appearance and disappearance of B_0_ cause the major shift in the RHEED patterns. When there is no other magnetic field source, B_0_ is the only factor that causes the shift in RHEED patterns, as in the case of the bare GaN sample. However, I_R_ and B_R_ will be induced on the InGaN nanoring surface, and B_R_ will also contribute to B_tol_. Thus, in this case, the movement of the RHEED patterns also presents the influence of B_R_.

## 4. Conclusions

In this study, the InGaN nanorings produced a magnetic field and generated ring currents in the nanoscale. Based on this nanostructure and phenomenon, a variety of nanodevices and nanosystems can be built, which can work under either active or passive modes, serving various functionalities. In the active mode, i.e., when the ring current is artificially injected, the nanorings can produce a magnetic field that is concentrated in the ring. In this case, the nanorings could work as a nanotransmitter, which can transfer energy over a space to a receiver through its magnetic field. If the receiver consists of multiple-ring structures, then the whole nanosystem can work as a nanotransformer. In the passive mode, i.e., when the ring current is induced by an external magnetic field, the nanoring arrays could be used as nanoscale sensors, transducers and resonators. Semiconductor nanorings can be used in magnetic field sensors to provide highly sensitive magnetic field detection capabilities in fields such as biomedicine and environmental monitoring. Furthermore, by combining semiconductor nanorings with other materials, magnetic nanocomposites with special properties can be developed, making them suitable for a variety of functional applications.

The demonstration of uniform and periodic InGaN nanoring arrays by NSAE presented here should be universal and transferable to other materials and structures [35,36]. This successful demonstration of electromagnetic induction based on nanorings may not only benefit the fundamental study of quantum effects but also open a way for energy conversion, transfer and detection in nanosystems.

## Data Availability

All the data, theory details, and simulation details that support the findings of this study are available from the corresponding authors upon reasonable request.

## Supplementary Materials

The following supporting information can be downloaded at: https://www.mdpi.com/article/10.3390/nano15030245/s1, Figure S1: Formation of InGaN nanorings; Figure S2: The sample structure, cathode luminescence (CL) experiment and CL results for the investigation of indium accumulation effect; Video S1: The RHEED pattern movements on the bare GaN surface when the external magnetic field is applied. The patterns monotonously move to another position; Video S2: The RHEED pattern movements on the InGaN nanoring surface when the external magnetic field is applied. There are several rebounds during the pattern shift; Video S3: The RHEED pattern movements on the InGaN nanoring surface when the external magnetic field is removed. There also are several rebounds during the pattern shift.
