# Supplementary material for: Electromagnetic Nanocoils Based on InGaN Nanorings
Source: Nanomaterials (Basel). 2025 Feb 5;15(3):245. doi: 10.3390/nano15030245 (PMC11820529; doi:10.3390/nano15030245)
Supplement: Supplementary file 1 [file nanomaterials-15-00245-s001.zip › nanomaterials-3427819-supplementary/nanomaterials-3427819-supplementary.pdf]

Supplementary Materials

Electromagnetic Nanocoils Based on InGaN Nanorings

Ziwen Yan [1], Peng Chen [1,*], Xianfei Zhang [1], Zili Xie [1], Xiangqian Xiu [1], Dunjun Chen [1], Hong Zhao [1], Yi Shi [1], Rong Zhang [2] and Youdou Zheng [1]

[1] Key Laboratory of Advanced Photonic and Electronic Materials, School of Electronic Science and Engineering, Nanjing University, Nanjing 210093, China; dg21230063@smail.nju.edu.cn (Z.Y.); 602022230055@smail.nju.edu.cn (X.Z); xzl@nju.edu.cn (Z.X.); xqxiu@nju.edu.cn (X.X.); djchen@nju.edu.cn (D.C.); zhaohong@nju.edu.cn (H.Z.); yshi@nju.edu.cn (Y.S.); ydzheng@nju.edu.cn (Y.Z.)
[2] School of Electronic Science and Technology, Xiamen University, Fujian, Xiamen, 361005, China; rzhang@nju.edu.cn
* Correspondence: pchen@nju.edu.cn

I. Atom migration on the surface leading to the nanoring formation

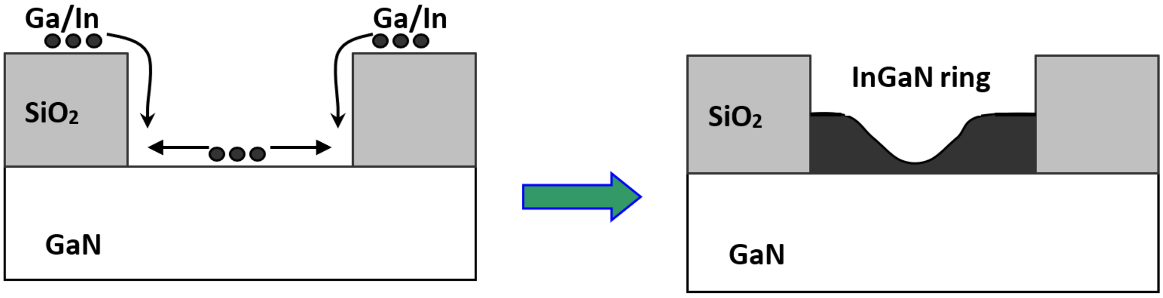

Figure S1. Formation of InGaN nanorings. Due to the difference in surface properties, InGaN cannot nucleate and grow on the SiO_2 surface. Some Ga/In adatoms will migrate to the GaN surface. At the same time, some of the adatoms at the hole's center area also tend to migrate to the edge because of lower surface potential at the edge areas. Finally, there are more Ga/In adatoms at the edge areas than at the center area. The local growth rate is faster at the edge areas than at the center areas, resulting in higher indium composition at the edge areas. In proper growth duration, the InGaN nanorings are formed [1,2].

II. Indium accumulation effect on the selective area growth.

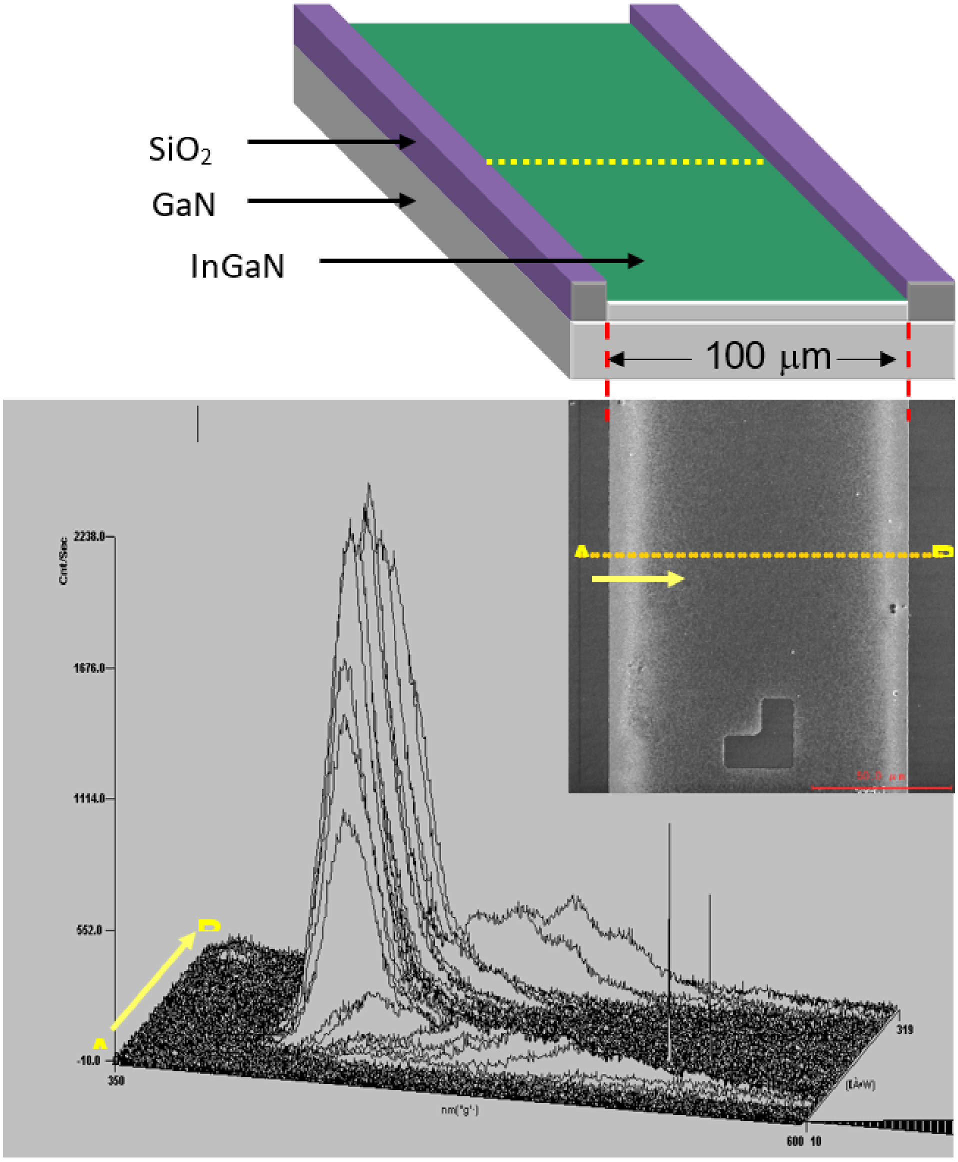

Figure S2. The sample structure, cathodeluminescence (CL) experiment and CL results for the investigation of indium accumulation effect.

An InGaN strip was grown with SiO_2 as the growth mask. Its thickness was 15 nanometers. Room-temperature cathode luminescence (CL) was carried out to measure the local emission across the InGaN strip point by point. The local CL spectra of the InGaN stripe show that the emission wavelengths from the edge areas (>550 nm) are much longer than those from the centre areas (420 nm). This result clearly reveals that indium composition is not uniform in the selective area growth of InGaN. The indium accumulation at the edge areas is severe and results in higher Indium compositions.

III. Video clips of the RHEED pattern movements under the external magnetic field. (Uploaded separately)

Chen-Video-1-
GaN surface.wmv

Video 1: The RHEED pattern movements on the bare GaN surface when the external magnetic field is applied. The patterns monotonously move to another position.

Chen-Video-2-
nanoring surface.[illegible]

Video 2: The RHEED pattern movements on the InGaN nanoring surface when the external magnetic **field is applied**. There are several rebounds during the pattern shift.

Chen-Video-3-
nanoring surface

Video 3: The RHEED pattern movements on the InGaN nanoring surface when the external magnetic field **is removed**. There also are several rebounds during the pattern shift.

References

1. Chen, P.; Chua, S.J.; Wang, Y.D.; Sander, M.D.; Fonstad, C.G. InGaN nanorings and nanodots by selective area epitaxy. *Appl. Phys. Lett.* **2005**, *87*, https://doi.org/10.1063/1.2056584.
2. Chen, A.; Chua, S.J.; Chen, P.; Chen, X.Y.; Jian, L.K. Fabrication of sub-100 nm patterns in SiO_2 templates by electron-beam lithography for the growth of periodic III–V semiconductor nanostructures. *Nanotechnology* **2006**, *17*, 3903–3908, https://doi.org/10.1088/0957-4484/17/15/048.
